# Construction and Validation of a Risk Prediction Model for Ventilator-Associated Pneumonia in Mechanically Ventilated Pediatric Patients

**DOI:** 10.1155/jonm/4445843

**Published:** 2025-10-28

**Authors:** Linxi He, Dong Ma, Yuanyuan Fu, Yang Gao, Yang Li, Jiaxin Yan, Yanping Liu

**Affiliations:** Department of Paediatric Intensive Care Unit, Shengjing Hospital of China Medical University, Shenyang, Liaoning, China

**Keywords:** mechanical ventilation, pediatrics, risk prediction model, ventilator-associated pneumonia

## Abstract

**Aim:**

This study identified risk factors for ventilator-associated pneumonia (VAP) in mechanically ventilated (MV) children and developed a risk prediction model to guide precision nursing interventions.

**Background:**

MV supports critically ill pediatric patients by improving oxygenation but may induce lung injury and increase VAP incidence.

**Methods:**

We retrospectively analyzed pediatric MV patients admitted to the Pediatric Intensive Care Unit (PICU) at Shengjing Hospital, China Medical University. Independent VAP risk factors were identified using binary logistic regression, and a prediction model was developed/validated with R software.

**Results:**

Absence of early enteral nutrition (EEN), duration of MV, frequency of endotracheal suctioning, central venous catheterization, and the types of antibiotics used were independent VAP risk factors (*p* < 0.05). The model demonstrated strong discrimination, with ROC-AUCs of 0.870 (95% CI: 0.816–0.924) and 0.761 (95% CI: 0.653–0.868) for derivation and validation cohorts, respectively. Hosmer–Lemeshow tests confirmed calibration (*p*=0.970 and *p*=0.524).

**Conclusions:**

This validated model effectively stratifies VAP risk in MV children, enabling early identification of high-risk patients and facilitating targeted nursing strategies.

**Implications for Nursing Management:**

The model allows rapid clinical screening for high-risk pediatric VAP cases. Interventions focused on modifiable risk factors may reduce VAP incidence.

## 1. Introduction

Mechanical ventilation (MV) is a cornerstone therapy in Pediatric Intensive Care Units (ICUs) (PICUs), providing vital respiratory support for critically ill children. By improving oxygenation and stabilizing hemodynamics, MV significantly reduces mortality [1, 2]. However, this life-sustaining intervention carries substantial risks, including increased intrathoracic pressure, ventilator-induced lung injury, and compromised pulmonary defenses, which collectively predispose patients to infections [3].

In pediatric MV patients, artificial airways disrupt upper respiratory tract barriers and impair mucociliary clearance, facilitating pathogenic colonization of the lower airways. This pathophysiology underlies ventilator-associated pneumonia (VAP), which affects 9%–68% of MV children with associated mortality rates of 24%–76% [4, 5]. Critically, VAP risk escalates by 1%–3% per additional day of MV [5], underscoring the imperative for early risk assessment and intervention.

VAP ranks among the most prevalent healthcare-associated infections in PICUs, second only to bloodstream infections. It represents the second most common hospital-acquired infection and the second most frequent healthcare-associated infection in children [6–8]. As per the latest guidelines from the Centers for Disease Control and Prevention's (CDC) National Healthcare Safety Network (NHSN), VAP is defined as pneumonia developing after > 2 consecutive days of MV [9]. Early-onset VAP should be suspected in pediatric patients undergoing invasive MV for > 48 h with evidence of lower respiratory tract microbial invasion [10]. VAP may prolong MV duration, drive the emergence of multidrug-resistant organisms, and significantly increase mortality, collectively contributing to adverse clinical outcomes [11]. Consequently, VAP risk prediction is critical, enabling clinicians to apply targeted interventions stratified by risk level, guide clinical decision-making, and ultimately reduce VAP incidence.

Current research primarily focuses on the epidemiology and risk factor analysis of VAP. Risk prediction models, serving as preventive and prognostic tools, can inform early warning systems and intervention strategies [12]. Consequently, implementing stratified risk management for MV pediatric patients carries significant clinical value. This approach enhances the identification of VAP-associated risk factors, enables targeted preventive interventions, and ultimately reduces VAP incidence. This study aims to develop and validate a VAP risk prediction model for MV pediatric patients, providing evidence-based clinical guidance.

## 2. Materials and Methods

### 2.1. Design and Sample

This retrospective study enrolled pediatric patients receiving invasive MV in the PICU of Shengjing Hospital, China Medical University, between January 2019 and March 2024. Participants met the following criteria:

  Inclusion criteria: ① Patients who received continuous MV for ≥ 48 h during their stay in the PICU of our hospital. ② Age between 1 and 14 years.
  Exclusion criteria: ① Patients with pulmonary infection before MV or within 48 h after the initiation of MV. ② Patients who were discharged, transferred, or died within 48–72 h after the initiation of MV. ③ Patients with incomplete clinical data. ④ Patients who had undergone endotracheal intubation and MV before admission.

### 2.2. Clinical Diagnostic Criteria for VAP

The diagnostic criteria for VAP were determined based on the CDC score [9]. VAP is defined as pneumonia occurring in patients who have been intubated or undergone tracheostomy and have received MV for more than 48 h. Pneumonia that develops within 48 h after weaning from MV or extubation is also considered VAP. Diagnostic criteria include the presence of fever (> 38.0°C) or hypothermia (< 36.0°C); leukopenia (≤ 4000 WBC/mm^3^) or leukocytosis (≥ 15,000 WBC/mm^3^); newly developed or changed purulent sputum, increased respiratory secretions or increased need for suctioning; new or worsening cough, dyspnea or tachypnea; rales or bronchial breath sounds; and worsening gas exchange. Radiological criteria include findings in patients with underlying disease (requiring at least two imaging results) or in otherwise healthy patients (requiring at least one imaging result) and meeting any of the following: new and persistent or progressive and persistent infiltrates, consolidation, cavitation, or pneumatoceles (in patients ≤ 1 year old).

### 2.3. Research Tools

Based on a review of domestic and international literature [13–15], and with a focus on selecting variables that align with the assessment content, we prioritized clinically relevant, quantifiable, and easily accessible indicators. After consulting with one chief intensivist, one associate chief intensivist, one associate chief nurse, and two senior nurses, we independently designed the “Risk Factors Survey for VAP in MV Pediatric Patients” based on expert opinions. After further discussion, we finalized the variables and classification methods, ultimately including 14 variables divided into three categories.

#### 2.3.1. General Information

This category includes gender, age, number of clinical diagnoses, Pediatric Critical Illness Score (PCIS) score, and whether early enteral nutrition (EEN) was administered. The PCIS involves evaluating 11 indicators: heart rate, blood pressure, respiration, arterial oxygen pressure, pH value, sodium, potassium, serum creatinine, blood urea nitrogen, hemoglobin, and gastrointestinal function. The total score is 100, with > 80 points indicating noncritical, 71–80 points indicating critical, and ≤ 70 points indicating extremely critical conditions. It has been validated to have good predictive value for assessing mortality risk [16]. EEN refers to the initiation of nutritional support via the gastrointestinal tract within 24–48 h of PICU admission. This approach aims to meet the nutritional needs of the patient as early as possible, supporting metabolic and immune function [17]. In this study, EEN specifically refers to enteral nutrition administered via a nasogastric tube.

#### 2.3.2. Invasive Procedures

This includes the duration of MV, frequency of endotracheal suctioning, number of intubations, use of parenteral nutrition, bronchoscopy, placement of central venous catheters, and whether the patient received a blood transfusion. Based on literature review [18] and expert consultation, the duration of MV was categorized into 2–7 days and > 7 days. The frequency of endotracheal suctioning was classified based on the number of times suctioning occurred every 3 h in relation to the duration of MV, divided into 0–56 times and > 56 times.

#### 2.3.3. Medication Use

This includes the number of antibiotics used and the number of sedatives administered, with three types used as the cutoff for classification.

### 2.4. Statistical Methods

First, data analysis was performed using SPSS 26.0 software. Categorical variables were described using frequency and percentage, and the chi-square (*X*^2^) test was used to compare differences between groups. A significance level of *p* < 0.05 was set to enhance sensitivity and avoid overlooking significant factors [19]. VAP infection status (no VAP infection = 0; VAP infection = 1) was used as the dependent variable, and variables with statistically significant differences in univariate analysis were used as independent variables in a binary logistic regression analysis to identify risk factors for VAP. A *p* value of < 0.05 (two-tailed test) was considered statistically significant. The identified risk factors from the regression analysis were used to create a nomogram using R software.

The model was validated using the following methods [20]: the area under the ROC curve (AUC), sensitivity, and specificity were used to evaluate predictive accuracy; an *AUC* > 0.7 indicates good predictive ability. The calibration curve and the Hosmer–Lemeshow test were used to assess the model's calibration and goodness of fit. A *p* value > 0.05 indicates a good fit. A calibration curve close to the reference line indicates good model calibration. Finally, clinical decision curve analysis was used to assess the clinical utility of the nomogram. The optimal region is where the curve lies above the two extreme curves in the upper right, indicating that the model has good clinical applicability.

## 3. Results

### 3.1. Basic Characteristics

The majority of MV pediatric patients were male, accounting for 55% (115/209) of the sample; most were aged between 1 and 5 years, representing 53.1% (111/209) of the sample. The number of clinical diagnoses was generally capped at five. Only 16.3% (34/209) of the patients underwent more than one intubation, and the majority of 77% (161/209) did not undergo bronchoscopy. Further details on general characteristics are provided in Table 1.

### 3.2. Risk Factor Screening for VAP

Univariate analysis results indicated that age, EEN, days of MV, endotracheal suctioning, tracheal intubations, parenteral nutrition, bronchoscopy, central venous catheter, blood transfusion, the variety of antibiotics used, and the variety of sedatives used showed statistically significant differences (*p* < 0.05). Other variables did not show statistically significant differences (*p* ≥ 0.05), as detailed in Table 1.

The results of the binary logistic regression analysis revealed that EEN, days of MV, endotracheal suctioning, central venous catheterization, and the variety of antibiotics used were independent risk factors for VAP (*p* < 0.05). Further details are provided in Table 2.

### 3.3. Construction of the VAP Risk Prediction Model

**Figure 1:**
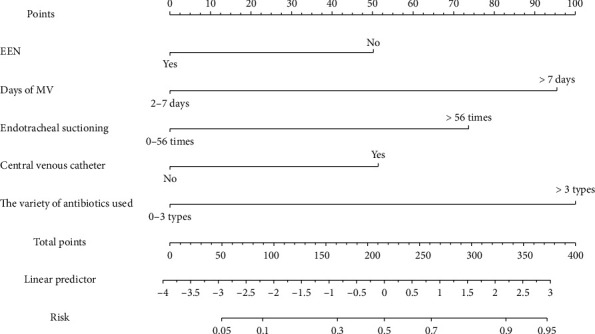
Risk prediction model for VAP.

Based on the results of the binary logistic regression analysis, the formula for the risk prediction model is as follows: *Z* = 1.088 (95% CI: 1.162–7.580) × EEN (no) + 1.510 (95% CI: 1.784–11.4774) × days of MV (> 7 days) + 1.369 (95% CI: 1.084–14.253) × endotracheal suctioning (> 56 times) + 1.196 (95% CI: 1.131–9.673) × central venous catheterization (yes) + 1.471 (95% CI: 1.295–14.639) × the variety of antibiotics used (> 3) − 4.769. Based on these risk factors, a nomogram was developed to predict the occurrence of VAP in MV pediatric patients, as shown in Figure 1.

### 3.4. Validation of the VAP Risk Prediction Model

**Figure 2:**
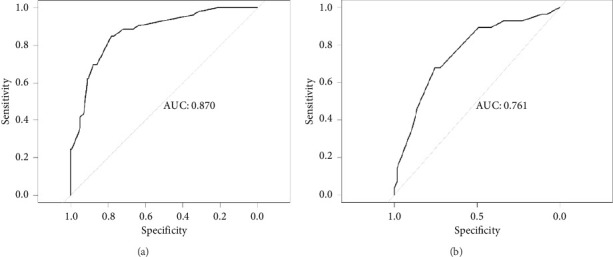
ROC curves for (a) development set and (b) validation set.

The discriminatory ability of the nomogram was evaluated using the ROC curve, as shown in Figure 2. The AUC for the modeling group was 0.870 (95% CI: 0.816–0.924), with a sensitivity of 0.849 and a specificity of 0.782. For the validation group, the AUC was 0.761 (95% CI: 0.653–0.868), with a sensitivity of 0.679 and a specificity of 0.754, indicating good discriminatory power of the model.

**Figure 3:**
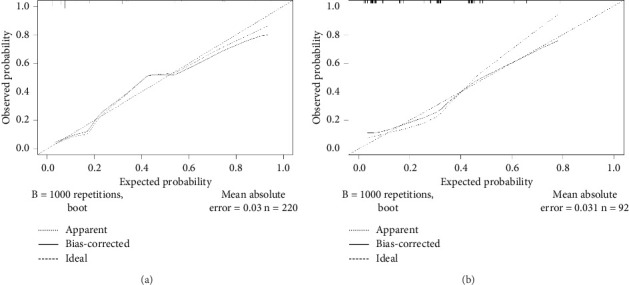
Calibration curves for (a) development set and (b) validation set.

The Hosmer–Lemeshow test results were *X*^2^ = 0.062, *p*=0.970 for the modeling group, and *X*^2^ = 1.292, *p*=0.524, for the validation group. The calibration curves for both groups are close to the reference line, as shown in Figure 3, demonstrating good calibration and goodness of fit for the prediction model.

**Figure 4:**
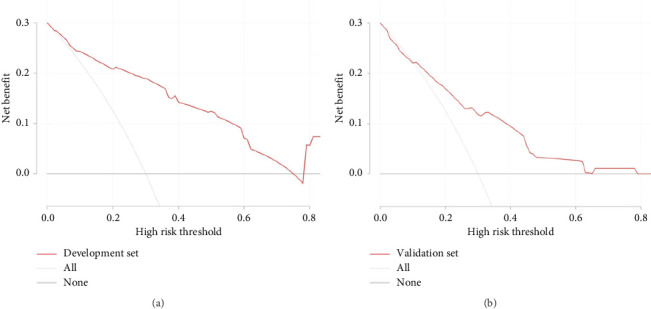
Clinical decision curves for (a) development set and (b) validation set.

Clinical decision curve analysis was used to evaluate the clinical utility of the nomogram. The results for the modeling group showed that the model provided a positive net benefit when the threshold range was between 0 and 0.78. In the validation group, the model demonstrated a positive net benefit when the threshold range was between 0 and 0.8, as illustrated in Figure 4. This indicates that the model has good clinical applicability.

## 4. Discussion

Patients on MV are at high risk of developing pneumonia and other pulmonary complications [21]. As a common and serious complication in MV patients, VAP is considered one of the most prevalent nosocomial infections in ICUs. Therefore, this study places significant emphasis on understanding and preventing VAP. Due to immature development of respiratory and immune systems in children, along with anatomical factors including narrower bronchi and fragile mucosa, pediatric patients are particularly susceptible to VAP, which can lead to increased mortality [14]. Consequently, healthcare providers should prioritize VAP management through early screening and prevention to reduce its incidence. This study developed an intuitive, clinically applicable VAP risk prediction tool that enables healthcare providers to accurately assess risk, which is crucial for timely intervention and improved patient outcomes.

The results indicate that the absence of EEN constitutes a significant risk factor for VAP in MV pediatric patients. Nutritional support plays a crucial role in treating ICU patients requiring MV. EEN aims not only to improve nutritional status but also to enhance immune regulation, prevent early gut bacterial translocation, and ultimately reduce VAP incidence [22]. Therefore, initiating EEN in pediatric patients without contraindications is essential, with continuous monitoring of nutritional status and tolerance to ensure adequate nutritional support.

Among invasive procedures, MV duration, endotracheal suctioning frequency, and central venous catheter presence were identified as VAP risk factors. These procedures represent common pathogen entry routes. Prolonged MV duration correlates with VAP risk [23, 24], as pathogens colonize ventilator circuits and lungs over time. Thus, closely monitoring vital signs, titrating oxygen support, and improving autonomous breathing capacity are critical. MV should be discontinued promptly when weaning criteria are met.

Endotracheal suctioning causes mechanical friction that dislodges bacterial biofilms, promoting tracheobronchial colonization [24]. Avoiding nonessential suctioning is imperative. Suction frequency should be individualized based on sputum characteristics and clinical status to minimize bacterial introduction and contamination risk.

Central venous catheters compromise natural defenses, enabling microbial invasion [25]. Frequent clinical contact during catheter care increases colonization risk. Meticulous maintenance and strict disinfection protocols during central venous procedures are essential for infection prevention.

In terms of medication use, the use of three or more types of antibiotics is associated with an increased risk of VAP. Broad-spectrum antibiotic overuse promotes multidrug-resistant strains, microbial imbalance, and opportunistic infections [26, 27]. Therefore, it is crucial to exercise caution in the selection of antibiotics, carefully considering the dosage, duration of treatment, and the potential for resistance. This approach can help minimize the risk of VAP and ensure more effective management of antibiotic therapy.

Recent research by Girona-Alarcón et al. [6] developed a pediatric VAP risk score based on gender, MV ≥ 4 days, PICU stay ≥ 7 days, and prior colonization. Our study additionally identifies EEN absence, suctioning frequency, central venous catheters, and multiantibiotic use as significant predictors, offering new intervention perspectives. While Chanci et al. [28] created an intubation timing model using machine learning, our model specifically predicts pediatric VAP risk during MV, enabling screening and personalized interventions.

Our risk prediction model demonstrated good discrimination (AUC > 0.7), calibration (Hosmer–Lemeshow *p* > 0.05), and clinical utility (positive net benefit on decision curve analysis). This provides an accurate tool for identifying high-risk patients. Effective VAP reduction requires early identification of at-risk children and targeted interventions. Our visual nomogram incorporating five key risk factors enables clinicians to intuitively assess VAP risk and implement timely prevention.

This study also has certain limitations. First, the relatively small sample size may affect the generalizability of the findings. Although the model demonstrated good predictive ability, further validation with larger sample sizes and a broader geographic scope is necessary to confirm the model's effectiveness. In addition, the variables included in this study may not be comprehensive. Future research should consider incorporating more relevant variables as the sample size expands to determine if any potentially predictive factors were overlooked. Finally, it is planned to validate the model with data from other hospital multicenters.

## 5. Conclusion

This study identified the absence of EEN, duration of MV, frequency of endotracheal suctioning, central venous catheterization, and types of antibiotics as risk factors for VAP in MV pediatric patients. By incorporating these variables, a risk prediction model was developed, providing clinical healthcare providers with an effective and straightforward tool to identify high-risk children for VAP. The risk prediction model has important clinical significance for the precise nursing and prevention of VAP in pediatric MV patients and has good clinical promotion value.

## 6. Implications for Nursing Management

Nursing managers can predict the risk of VAP by a nomogram and manage the patients according to the risk probability, so as to realize early detection, diagnosis, and treatment. Depending on the selected risk factors, more precise interventions will be developed for different children to reduce the incidence of VAP.

## Figures and Tables

**Table 1:** Comparison of VAP incidence among MV children by different characteristics.

Variable	Total	No VAP	VAP	*X* ^2^	*p*
Gender				2.390	0.122
Male	115 (55)	81 (52)	34 (64)		
Female	94 (45)	75 (48)	19 (36)		
Age				9.501	**0.009**
1–5 years	111 (53)	92 (59)	19 (36)		
6–10 years	58 (28)	40 (26)	18 (34)		
11–14 years	40 (19)	24 (15)	16 (30)		
Number of diagnoses				3.480	0.062
0–5 types	94 (45)	76 (49)	18 (34)		
> 5 types	115 (55)	80 (51)	35 (66)		
PCIS score				1.123	0.551
Noncritical	155 (74)	115 (74)	40 (75)		
Critical	46 (22)	36 (23)	10 (19)		
Extremely critical	8 (4)	5 (3)	3 (6)		
EEN				10.623	**0.001**
No	72 (34)	44 (28)	28 (53)		
Yes	137 (66)	112 (72)	25 (47)		
Days of MV				51.331	**< 0.001**
2–7 days	145 (69)	129 (83)	16 (30)		
> 7 days	64 (31)	27 (17)	37 (70)		
Endotracheal suctioning				20.200	**< 0.001**
0–56 times	68 (32)	64 (41)	4 (7)		
> 56 times	141 (68)	92 (59)	49 (93)		
Tracheal intubation				10.102	**0.001**
Once	175 (84)	138 (88)	37 (70)		
More than once	34 (16)	18 (12)	16 (30)		
Parenteral nutrition				17.256	**< 0.001**
No	181 (87)	144 (92)	37 (70)		
Yes	28 (13)	12 (8)	16 (30)		
Bronchoscopy				4.853	**0.028**
No	161 (77)	126 (81)	35 (66)		
Yes	48 (23)	30 (19)	18 (34)		
Central venous catheter				16.263	**< 0.001**
No	148 (71)	122 (78)	26 (49)		
Yes	61 (29)	34 (22)	27 (51)		
Blood transfusion				15.771	**< 0.001**
No	134 (64)	112 (72)	22 (41)		
Yes	75 (36)	44 (28)	31 (59)		
The variety of antibiotics used				33.188	**< 0.001**
0–3 types	182 (87)	148 (95)	34 (64)		
> 3 types	27 (13)	8 (5)	19 (36)		
The variety of sedatives used				5.491	**0.019**
0–3 types	96 (46)	79 (51)	17 (32)		
> 3 types	113 (54)	77 (49)	36 (68)		

*Note:p* < 0.05 is shown in bold.

**Table 2:** Binary logistic regression analysis for VAP in MV children.

Variable	*β*	SE	Wald *X*^2^	*p*	OR	95% CI
Age						
1–5 years (reference)						
6–10 years	0.417	0.500	0.693	0.405	1.517	0.569–4.044
11–14 years	0.299	0.561	0.284	0.594	1.348	0.449–4.045
EEN						
Yes (reference)						
No	1.088	0.478	5.168	**0.023**	2.968	1.162–7.580
Days of MV						
2–7 days (reference)						
> 7 days	1.510	0.475	10.102	**0.001**	4.525	1.784–11.477
Endotracheal suctioning						
0–56 times (reference)						
> 56 times	1.369	0.657	4.338	**0.037**	3.931	1.084–14.253
Tracheal intubation						
Once (reference)						
More than once	0.822	0.529	2.411	0.120	2.274	0.806–6.414
Parenteral nutrition						
No (reference)						
Yes	0.291	0.635	0.211	0.646	1.338	0.386–4.645
Bronchoscopy						
No (reference)						
Yes	0.694	0.522	1.771	0.183	2.002	0.720–5.568
Central venous catheter						
No (reference)						
Yes	1.196	0.547	4.777	**0.029**	3.308	1.131–9.673
Blood transfusion						
No (reference)						
Yes	−0.067	0.528	0.016	0.899	0.935	0.332–2.634
The variety of antibiotics used						
0–3 types (reference)						
> 3 types	1.471	0.619	5.656	**0.017**	4.354	1.295–14.639
The variety of sedatives used						
0–3 types (reference)						
> 3 types	0.540	0.446	1.467	0.226	1.717	0.716–4.115
Constant	−4.769	0.775	37.843	< 0.001	0.008	

*Note:* Variable coding: number of diagnoses: 0–5 types = 0, > 5 types = 1; EEN: no = 0, yes = 1; days of MV: 2–7 days = 0, > 7 days = 1; endotracheal suctioning: 0–56 times = 0, > 56 times = 1; tracheal intubations: once = 0, more than once = 1; parenteral nutrition: no = 0, yes = 1; bronchoscopy: no = 0, yes = 1; central venous catheter: no = 0, yes = 1; blood transfusion: no = 0, yes = 1; the variety of antibiotics used: 0–3 types = 0, > 3 types = 1; the variety of sedatives used: 0–3 types = 0, > 3 types = 1. *p* < 0.05 is shown in bold.

## Data Availability

The data that support the findings of this study are available on request from the corresponding author. The data are not publicly available due to privacy or ethical restrictions.
